# Safety of pulsed field ablation using a variable loop circular catheter in a patient with a cardiac implantable electronic device

**DOI:** 10.1016/j.hrcr.2024.10.018

**Published:** 2024-10-22

**Authors:** Salik ur Rehman Iqbal, Thomas Kueffer, Ajay Panakal, Laurent Roten, Tobias Reichlin

**Affiliations:** 1 Department of Cardiology, Inselspital, Bern University Hospital, University of Bern, Bern, Switzerland; 2 Johnson & Johnson MedTech, Zug, Switzerland

**Keywords:** CIED, Pulsed field ablation, Variable loop circular catheter, Pulmonary vein isolation, PFA catheters

**Key Teaching Points**

• Pulsed field ablation generates strong electrical fields and this can potentially result in cardiac implantable electronic device malfunction.
• Due to differences in pulsed field ablation waveforms and catheter designs, the safety of any new pulsed field ablation catheter system for use in patients with pacemakers and implantable cardioverter-defibrillators has to be studied.
• A novel variable loop circular pulsed field ablation catheter (VARIPULSE; Biosense Webster) was safely used for pulmonary vein isolation in a patient with an implanted dual-chamber pacemaker with no interactions observed.

## Introduction

Preclinical and clinical studies have demonstrated the safety and efficacy of a variable loop circular catheter (VLCC) for pulsed field ablation (PFA).^1^^,^^2^ There are a few studies exploring the safety of the pentaspline PFA catheter in patients with cardiac implantable electronic devices (CIEDs).^3^, ^4^, ^5^ The safety of the PFA VLCC in this common patient population is so far unexplored because they have been excluded from the clinical studies of the VLCC to date.

## Case report

A 76-year-old woman with a history of ischemic heart disease and a dual chamber pacemaker implanted because of paroxysmal atrioventricular block 2 years ago presented with symptomatic paroxysmal atrial fibrillation. Programmed in a DDD mode with a lower rate of 60 beats/min, the patient required <1% of right ventricular pacing. Transthoracic echocardiography showed a normal left ventricular ejection fraction, diastolic dysfunction grade II, and no valvular pathologies, but a significant dilatation of the left atrium (left atrial volume index, 50 mL/m^2^). The management options were discussed and the patient elected to undergo pulmonary vein isolation.

**Figure 1:**
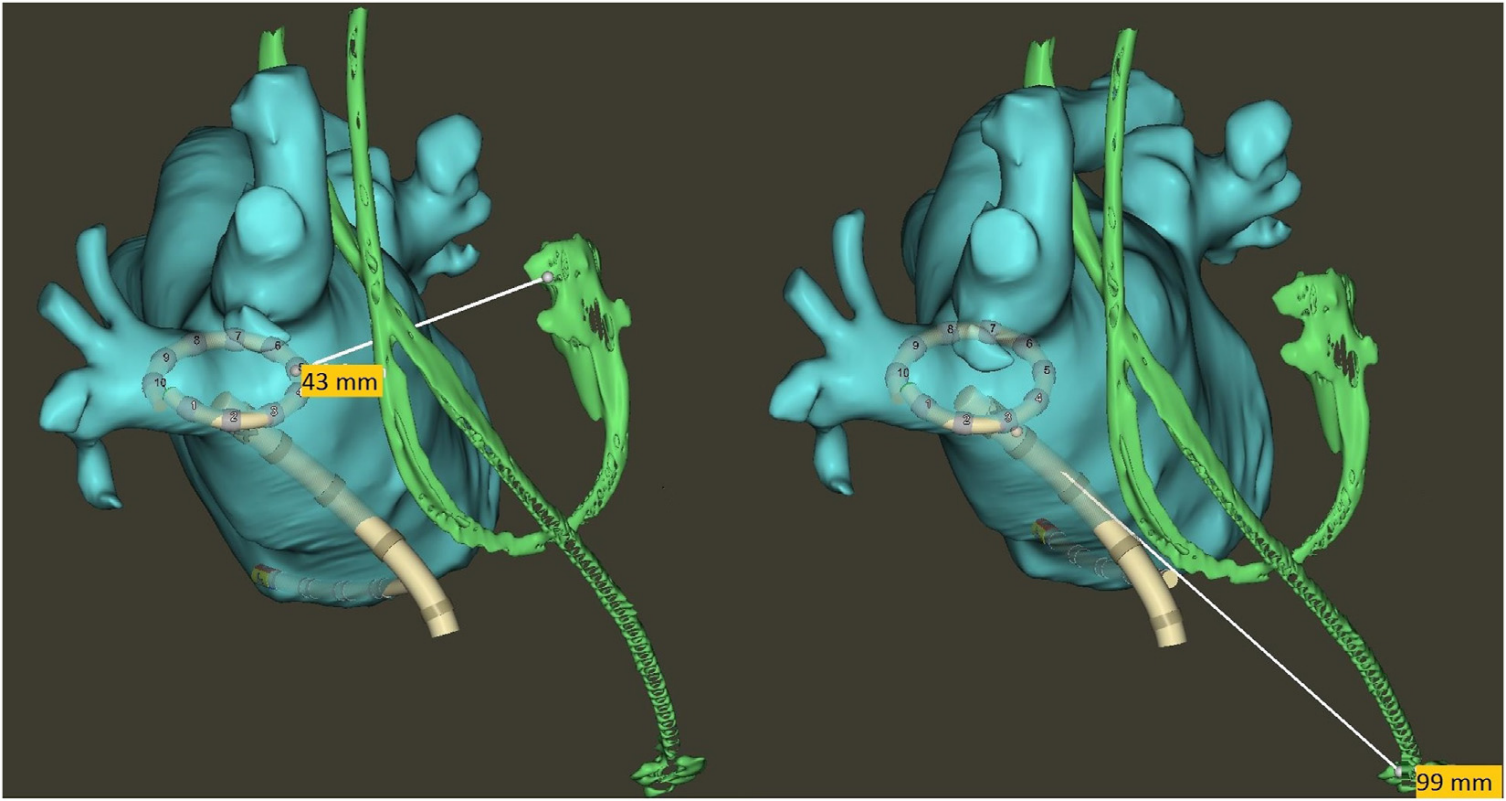
Computed tomography reconstruction of the left atrium and pacemaker leads demonstrating the proximity of the leads to the VARIPULSE pulsed field ablation catheter (Biosense Webster) in the right inferior pulmonary vein (right anterior oblique view). Distance to the right atrium (left) and right ventricle (right) lead tips measured is shown.

Before the procedure, transesophageal echocardiography and cardiac computed tomography were performed to exclude left atrial thrombi and to visualize the left atrial anatomy. Given that the patient had intrinsic atrioventricular conduction at the beginning of the procedure, the pacemaker was left in DDD mode. The procedure was performed under deep conscious sedation using midazolam, fentanyl, and propofol guided by a physician-led, nurse-administered protocol.^6^ A single transseptal puncture was performed. Heparin was given to achieve a target activated clotting time >350 seconds. Electroanatomic mapping of the left atrium was performed using an irrigated, bidirectional, multi-electrode (ie, 10 electrodes) PFA VLCC in conjunction with the CARTO 3 mapping system (VARIPULSE Platform; Biosense Webster). Image integration of the preprocedural computed tomography was performed (Figure 1) for procedural guiding and to visualize the pacemaker leads. Short-duration, high-voltage bipolar biphasic pulses of 1800 V were systematically applied to all 4 pulmonary veins on both ostial and antral positions using the TRUPULSE Generator (Biosense Webster), according to a protocol published previously.^7^^,^^8^ The left superior pulmonary vein required additional ablations due to residual signals. A total of 24 ablations (72 PFA applications in total) were performed. Subsequent remapping of the left atrium and the pulmonary veins with the PFA VLCC confirmed the successful isolation of all pulmonary veins, as evidenced by both entrance and exit block. The procedure duration was 93 minutes.

Pacemaker interrogation at the end of the procedure revealed unchanged and normal values for sensing, pacing, and impedance compared with before ablation, and no programming changes were made. The patient was discharged home the following day. At the 3-month follow-up visit in the outpatient clinic, the patient reported disappearance of any symptoms related to atrial fibrillation. Pacemaker parameters were normal with preserved battery longevity. Interrogation of the device memory revealed a single 21-minute episode of atrial fibrillation on day 54 after the ablation.

## Discussion

PFA is a novel, nonthermal ablation technology that promises improved safety due to its tissue preferentiality, while maintaining efficacy.^1^^,^^2^^,^^9^^,^^10^ The strong electrical field generated by the PFA catheter can potentially result in CIED malfunction, including damage to the electrical circuitry as well as inappropriate response to sensed electrical pulses, potentially leading to pacing inhibition, inappropriate implantable cardioverter-defibrillator therapy, and/or mode switches.^4^ This raised concerns about potential electrical interference with, and subsequent malfunction of, CIEDs after PFA procedures. Accordingly, patients with implanted CIEDs have been excluded from the investigational device exemption trials with the PFA VLCC.^1^^,^^2^^,^^11^

There is emerging evidence for the safety of PFA in the presence of CIEDs when using a pentaspline PFA catheter. Chen et al^5^ reported on the safety of the pentaspline catheter in a study of 20 patients with transvenous CIEDs. Antitachycardia therapies were turned off for implantable cardioverter-defibrillators, but device modes were kept unchanged. Eight of the 20 patients were pacemaker-dependent and, despite PFA applications, no pacing inhibition was seen. The study included patients for pulmonary vein isolation only.^5^ These initial results were confirmed in 2 subsequent small case series using the pentaspline PFA catheter.^3^^,^^4^

Different PFA catheter systems are remarkably different with regard to the PFA waveform and the catheter design. The PFA waveforms are composed of a number of engineered factors, including pulse polarity, pulse length, pulse width, interphase pauses, number of pulses in a train, frequency, and voltage, among many other considerations. The catheter design, electrode size, electrode spacing, and shape provide additional complexity to how the waveform ultimately impacts the tissue, and could potentially interfere with adjacent metals.

As such, it is likely that each unique PFA device needs to be individually assessed to ensure it can be safely used in patients with implanted CIEDs. Table 1 provides a comparison of key characteristics of the PFA VLCC and the pentaspline PFA catheter. Although the waveform polarity and voltages are similar, other design features are either unknown or seemingly different (such as the number of electrodes and catheter shapes).

**Table 1:** Comparison of the 2 pulsed field ablation catheters

Characteristic	VARIPULSE (Biosense Webster)	FARAPULSE (Boston Scientific)
Catheter tip dimensions	Variable loop circular catheter with a diameter of 25–35 mm	Pentaspline catheter, interchangeable basket and flower configurations, available with 31-mm or 35-mm diameter
Electrode size, mm	3	2
No. of electrodes	10	20
Voltage, V	1800	2000
Waveforms	Biphasic, bipolar	Biphasic, bipolar
Pulse train details	Proprietary no. of pulses delivered within 250 ms	Proprietary no. of pulses delivered within 200 ms
No. of pulse trains per ablation	3	5
Length of pauses separating the pulse trains	10 s	300 ms

## Conclusion

Our case report is among the first to report on the use of the PFA VLCC in a patient with an implanted CIED. The ability of the PFA VLCC to perform both 3-dimensional electrophysiologic mapping, as well as delivery of PFA, could be of interest for patients with a CIED, as it allows single transseptal access and minimizes the need for catheter exchanges, which can be a concern in patients with multiple/recent leads. Although it is very reassuring that no interaction with the pacemaker of the patient was observed in this case, larger series are needed in patients, also including other types of CIEDs, such as implantable cardioverter-defibrillators or leadless pacemakers. This will also allow optimizing periprocedural device management with regard to the need for reprogramming CIEDs and brady- and tachy-therapies during PFA procedures using the PFA VLCC.

## Funding Sources

This research did not receive any specific grant from funding agencies in the public, commercial, or not-for-profit sectors.

## Disclosures

Laurent Roten: speaker honoraria from Abbott/SJM, consulting honoraria from Medtronic, and a research grant to the institution for an investigator-initiated trial from Medtronic. Tobias Reichlin: research grants from the Swiss National Science Foundation, the Swiss Heart Foundation, the sitem insel support funds, Biotronik, Boston Scientific, and Medtronic, all for work outside the submitted study. Speaker/consulting honoraria or travel support from Abbott/SJM, Bayer, Biosense-Webster, Biotronik, Boston-Scientific, Farapulse, Medtronic, and Pfizer-BMS, all for work outside the submitted study. Support for his institution’s fellowship program from Abbott/SJM, Biosense-Webster, Biotronik, Boston-Scientific and Medtronic for work outside the submitted study. Thomas Kueffer: grant from Swiss Heart foundation. Ajay Panakal is a New Technology specialist, Johnson & Johnson MedTech. Salik ur Rehman Iqbal has no conflicts of interest.
